# MutaNET: a tool for automated analysis of genomic mutations in gene regulatory networks

**DOI:** 10.1093/bioinformatics/btx687

**Published:** 2017-10-26

**Authors:** Markus Hollander, Mohamed Hamed, Volkhard Helms, Kerstin Neininger

**Affiliations:** 1 Center for Bioinformatics, Campus Building E2.1, Saarland University, Saarbrücken, Germany; 2 Institute for Biostatistics and Informatics in Medicine and Ageing Research, Rostock University Medical Center, Rostock, Germany; 3 Saarbrücken Graduate School of Computer Science, Saarland University, Saarbrücken, Germany

## Abstract

**Summary:**

Mutations in genomic key elements can influence gene expression and function in various ways, and hence greatly contribute to the phenotype. We developed *MutaNET* to score the impact of individual mutations on gene regulation and function of a given genome. *MutaNET* performs statistical analyses of mutations in different genomic regions. The tool also incorporates the mutations in a provided gene regulatory network to estimate their global impact. The integration of a next-generation sequencing pipeline enables calling mutations prior to the analyses. As application example, we used *MutaNET* to analyze the impact of mutations in antibiotic resistance (AR) genes and their potential effect on AR of bacterial strains.

**Availability and implementation:**

*MutaNET* is freely available at https://sourceforge.net/projects/mutanet/. It is implemented in Python and supported on Mac OS X, Linux and MS Windows. Step-by-step instructions are available at http://service.bioinformatik.uni-saarland.de/mutanet/.

**Supplementary information:**

Supplementary data are available at *Bioinformatics* online.

## 1 Introduction

Mutations can affect an organismal phenotype in many ways, whereby the genomic position of a variant is of fundamental importance. Coding mutations can influence protein function (Woodford and Ellington, 2007), whereas those in regulatory sites can affect expression of the gene itself and of genes in that regulatory cascade (Grkovic *et al.*, 2001). Thereby, gene expression levels are regulated by transcription factors (TFs) via binding to transcription factor binding sites (TFBS) (de Jong, 2002).

We developed *MutaNET* that scores the potential impact of mutations on gene expression and protein function of a given genome. *MutaNET* statistically compares the mutational impact on coding regions, promoters and TFBS using refined scoring schemes. If regulatory information is provided as well, a gene regulatory network (GRN) is constructed to examine the global effect of individual mutations. To the best of our knowledge, a similar tool that implements a combinatory analysis of variant calling, statistical analysis and incorporation of a GRN does not exist yet. Moreover, *MutaNET* supports statistical comparisons between different gene groups such as bacterial AR and non-AR genes. Since mutations in AR genes can cause or affect AR of bacterial strains, we used *MutaNET* for a detailed analysis of mutations in AR genes and their possible impact on AR.

## 2 MutaNET description

**Fig. 1:**
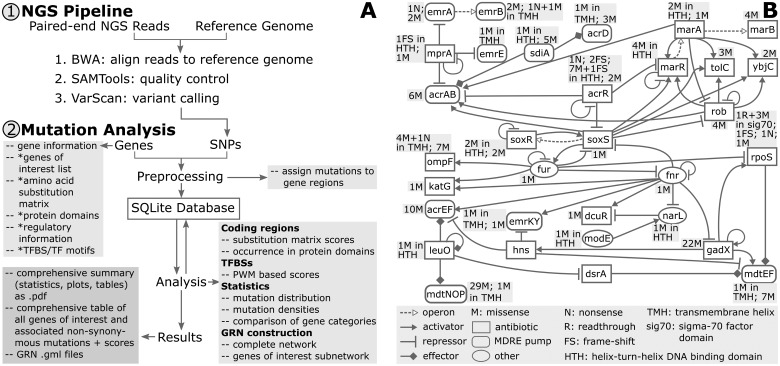
(**A**) *MutaNET* workflow (*optional). (**B**) Truncated AR regulatory subnetwork of *E.coli* K-12 strain provided by *MutaNET*. Since the development of AR can be based on an increased antibiotic efflux (Levy and Marshall, 2004), the AR genes of interest additionally comprise multidrug-resistant efflux (MDRE) pumps and their direct regulators

*MutaNET* consists of several analysis steps: a mutation calling pipeline, a statistical comparison of mutations in different genomic regions, and generation of the underlying GRN, see Figure 1A. Mutations can either be called automatically from NGS paired-end reads using the embedded mutation calling pipeline presented in Hamed *et al.* (2015), or mutations can be provided by the user. Mutations are then assigned to different genomic regions (coding region, promoter and TFBS) using in-house scripts analogous to BEDTools (Quinlan *et al.*, 2010). Statistically significant differences are identified based on the Wilcoxon rank-sum test. 

*MutaNET* differentiates between synonymous, missense, nonsense, readthrough and reading-frame shift mutations. The effect of mutations in coding regions is assessed using an amino acid (AA) substitution matrix and a pairwise sequence alignment between reference and mutated protein sequence, see Supplementary Material. Since the impact of a mutation is influenced by its position in the protein, protein domain information downloaded from UniProt (uniprot.org) is incorporated in the analysis as well. Mutations in TFBS can increase or decrease the ability of the corresponding regulator to bind (Grkovic *et al.*, 2001; Melton *et al.*, 2015). A score is computed that indicates whether mutations in TFBS are likely to increase or decrease the binding ability of the TF. This TFBS mutation score is based on a position weight matrix constructed from TF motif sequence alignments and a comparison between observed and random mutations following the method by Melton *et al.* (2015). A GRN is constructed to decipher the global effect of mutations. The nodes (genes) display the number of non-synonymous coding, promoter and TFBS mutations. This allows to quickly identify genes with mutations that directly or indirectly regulate specific genes, such as AR genes. GRNs can be further processed using programs such as Cytoscape (Smoot *et al.*, 2011).

## 3 Case study

To demonstrate one possible application, we applied *MutaNET* to *E.coli* K-12 and *S.aureus* NCTC 8325 reference strains. Mutations were called with the embedded NGS pipeline from a set of 300 *E.coli* (NCBI, see Supplementary Material) and 30 *S.aureus* strains (Hamed *et al.*, 2015). Regulatory and AR information was taken from RegulonDB (regulondb.ccg.unam.mx), AureoWiki (aureowiki.med.uni-greifswald.de), PATRIC (patricbrc.org) and the literature.

We report 93 204 and 18 447 mutations of which 3035 and 372 were found in AR genes for *E.coli* and *S.aureus*, respectively. For *S.aureus*, the number of missense mutations was significantly lower (*P* = 0.02) in AR genes (21.3%) compared to non-AR genes (28.4%). AR genes are important for survival of the strain and missense mutations in their key protein domains could decrease fitness. A more detailed report can be found in the Supplementary Material.

To analyze the global effect of mutations, an AR regulatory subnetwork of *E.coli* was constructed, see Figure 1B. The respective GRN for *S.aureus* is shown in the Supplementary Material. We found several severe mutations in the *E.coli* HTH domain of transcriptional regulator AcrR that could lead to malfunction. In consequence, the repression of *acrA* and *acrB* genes, which code for MDRE pump subunits, might be disturbed. This could lead to the development of AR due to over-expression of the MDRE pump. The *acrAB* operon is negatively regulated by repressor MprA for which a frame-shift mutation in the HTH domain and a missense mutation were observed. Dysfunction of MprA could lead to over-expression of multidrug transporters EmrA/B/E that confer AR.

Moreover, *MutaNET* reports several mutations in the genes *parC* and *gyrA* that are associated with AR. Interestingly, we observed these mutations for both *E.coli* and *S.aureus* proteins, see Supplementary Table S6 and Supplementary Figure S2, suggesting a similar AR mechanism.

## 4 Conclusion

The *MutaNET* software supports and facilitates the investigation of individual mutations in a given genome. The sequential analysis steps provide a detailed report of different mutation types in distinct genomic elements and also allows their statistical comparison between gene groups, such as AR and non-AR genes. Moreover, integration of the underlying GRN greatly helps in estimating the global impact of mutations on gene expression. Application of *MutaNET* to a resistance gene dataset considerably simplified the identification of candidate resistance mutations. It was also possible to decipher similar resistance mechanisms across species.

## Supplementary Material

Supplementary Data

## References

[btx687-B1] de Jong H. (2002) Modeling and simulation of genetic regulatory systems: a literature review. J. Comput. Biol., 9, 67–103. 11911796 10.1089/10665270252833208

[btx687-B2] Grkovic S. et al (2001) Transcriptional regulation of multidrug efflux pumps in bacteria. Semin. Cell Dev. Biol., 12, 225–237. 11428915 10.1006/scdb.2000.0248

[btx687-B3] Hamed M. et al (2015) Whole genome sequence typing and microarray profiling of nasal and blood stream methicillin-resistant *Staphylococcus aureus* isolates: clues to phylogeny and invasiveness. Infect. Genet. Evol., 36, 475–482. 26297907 10.1016/j.meegid.2015.08.020

[btx687-B4] Levy S.B., Marshall B. (2004) Antibacterial resistance worldwide: causes, challenges and responses. Nat. Med., 10, S122–129. 15577930 10.1038/nm1145

[btx687-B5] Melton C. et al (2015) Recurrent somatic mutations in regulatory regions of human cancer genomes. Nat. Genet., 47, 710–716. 26053494 10.1038/ng.3332 PMC4485503

[btx687-B6] Quinlan A. et al (2010) BEDTools: a flexible suite of utilities for comparing genomic features. Bioinformatics, 26, 841–842. 20110278 10.1093/bioinformatics/btq033 PMC2832824

[btx687-B7] Smoot M.E. et al (2011) Cytoscape 2.8: new features for data integration and network visualization. Bioinformatics, 27, 431–432. 21149340 10.1093/bioinformatics/btq675 PMC3031041

[btx687-B8] Woodford N., Ellington M.J. (2007) The emergence of antibiotic resistance by mutation. Clin. Microbiol. Infect., 13, 5–18. 17184282 10.1111/j.1469-0691.2006.01492.x

