# Supplementary material for: MutaNET: a tool for automated analysis of genomic mutations in gene regulatory networks
Source: Bioinformatics. 2017 Oct 26;34(5):864–6. doi: 10.1093/bioinformatics/btx687 (PMC6192214; doi:10.1093/bioinformatics/btx687)
Supplement: Supplementary Data [file btx687_supplement.pdf]

Supplementary Material

MutaNET: a tool for automated analysis of genomic mutations in gene regulatory networks

Markus Hollander, Mohamed Hamed, Volkhard Helms, Kerstin Neininger

1 NGS pipeline

The stand-alone NGS pipeline developed in our group was first applied in [1]. First, paired-end reads are mapped to the reference genome using the Burrows-Wheeler Alignment Tool [2]. Next, reads with low mapping quality (*Phred* quality score < 30) as well as duplicated PCR reads are removed using *SAMtools* [3, 4]. After sorting the final alignments, SNPs, and indels are called applying *VarScan2* [5].

2 Mutation analysis

Mutations are assigned to the genomic regions using in-house scripts analogous to BEDtools [6]. Genomic regions comprise coding region, promoter region, and TFBSs. Note that a mutation can be associated with multiple genes or genomic regions due to overlaps.

2.1 Coding regions

MutaNET differentiates between synonymous, missense, nonsense, readthrough, and reading-frame shift mutations. Since the position in the protein influences the impact of a mutation, protein domain(s) are incorporated in the analysis. The effect on the amino acid sequence is automatically assessed using a pairwise sequence alignment of the reference (R) and mutated (M) amino acid sequence together with an amino acid substitution matrix S (here: PAM10). The overall substitution score $\in [0, 1]$ is computed as

$$Score = \frac{\sum_{i=1}^{N} S_{R[i],M[i]} - \sum_{i=1}^{N} \min_{j \in AA} \{S_{R[i],j}\}}{\sum_{i=1}^{N} \max_{j \in AA} \{S_{R[i],j}\} - \sum_{i=1}^{N} \min_{j \in AA} \{S_{R[i],j}\}} \tag{1}$$

with the matrix entry $S_{R[i],M[i]}$ of the reference and mutated AA at sequence position i, and the aligned sequence length N. The lower the score, the more the mutated AA sequence deviates from the reference and the higher is likely the impact of the mutation.

2.2 Transcription factor binding sites

Mutations in transcription factor binding sites (TFBSs) can increase or decrease the ability of the corresponding transcription factor (TF) to interact with the respective gene [7, 8]. Depending on the effect of the TF on gene transcription, i.e. activation or repression, gene expression can be up– or downregulated.

MutaNET computes a score that indicates whether mutations in TFBSs are likely to increase or decrease the binding ability of the TF. This TFBS mutation score is based on position weight matrices (PWM) and the comparison between observed and random mutations. Our implementation follows the method reported by Melton *et al.* (2015) [8]. However, instead of using pre-computed PWMs from various databases, the PWMs are constructed by the *MutaNET* tool from TF motif sequence alignments provided as input.

For every TF with available motif sequence alignment, a PWM is computed. The PWM entries define the probability of a particular DNA base (rows) to occur at a certain motif position (columns). For each position in the motif alignment, the base counts are divided by the number of sequences in the alignment to obtain a position frequency matrix ($PFM_{k,i}$). To avoid zero entries, pseudocounts with a value of 0.8 are added to all entries [9]. It was suggested that integrating the conservation of a matrix position leads to a better performance of PWM–based matrix similarity scores [10]. Thus, we calculated analogously to [10] the conservation $C(i)$ of position i in the motif as

$$C(i) = \sum_{k \in \{A,C,T,G\}} PFM_{k,i} \times \log\left(\frac{PFM_{k,i}}{b}\right) \tag{2}$$

with the frequencies of the DNA bases k occurring at position i in the $PFM_{k,i}$ and the log likelihood transformed $PFM_{k,i}$. For simplicity, we assume that every base has the same frequency in the DNA and thus the background model was defined as $b = 0.25$. The final PWM is then calculated by multiplying the conservation level with the entries of the position frequency matrix:

$$PWM_{k,i} = C(i) \times PFM_{k,i} \tag{3}$$

The normalized TFBS score of a motif sequence S was then defined as

$$Score_{TFBS}(S) = \frac{\sum_{i=1}^{N} PWM_{S[i],i} - \sum_{i=1}^{N} \min_k \{PWM_{k,i}\}}{\sum_{i=1}^{N} \max_k \{PWM_{k,i}\} - \sum_{i=1}^{N} \min_k \{PWM_{k,i}\}} \tag{4}$$

with the sequence length N, the DNA bases $k \in \{A, C, T, G\}$, and the position $i \in \{1, ..., N\}$ in the motif alignment. If $Score_{TFBS} = 0$, this means that the sequence and the transcription factor motif have maximum distance. In contrast, if $Score_{TFBS} = 1$, this denotes that the sequence and the motif are highly similar.

$Score_{TFBS}(S)$ is then calculated for the reference (wild-type) binding site sequence, the observed mutated sequence, and randomly mutated binding sequences. To estimate a change in the TF binding ability, the difference between the score of the reference (wild-type) binding site and the score of the observed mutated binding site as well as the difference between the reference and the randomly mutated binding sites are calculated as

$$scoreMut = Score_{TFBS}(Ref) - Score_{TFBS}(Mut)$$

$$scoreRnd = \frac{1}{r} \times \sum_{i=1}^{r} (Score_{TFBS}(Ref) - Score_{TFBS}(R(i)))$$

with the TFBS sequence of the wild-type Ref, the observed mutated TFBS sequence Mut, and the i–th generated randomly mutated binding site $R(i)$ with $i \in \{1, ..., r\}$.

A score < 0 suggests that the mutated score is more similar to the motif than the reference sequence and therefore suggests an increased ability of the TF to bind to the mutated binding site. Similarly, a score > 0 suggests a decrease in the binding ability. If the score is 0 there is probably no change.

During randomization, the mutation type is preserved, i.e. if the observed mutation is a transversion then the random mutations are also transversions. The Wilcoxon rank–sum test [11] is then applied to analyze if there are significant differences between $scoreMut$ and $scoreRnd$ for mutations in antibiotic and non–antibiotic resistance genes.

3 Case studies

To demonstrate one possible usage, we applied *MutaNET* to the completely sequenced and annotated *E. coli* K-12 and *S. aureus* NCTC 8325 reference strains to analyze the bacterial genotype underlying antibiotic resistance. Paired-end reads were based on sequence type 131 (ST131) and clonal complex five (CC5) for *E. coli* and *S. aureus*, respectively [1, 12]. The *S. aureus* strains used here were studied in [1]. Genome sequences for *E. coli* ST131 were retrieved from the National Center for Biotechnology Information (NCBI) (https://www.ncbi.nlm.nih.gov/) with the following accession numbers (BioSample or BioProject): PRJNA383781, PRJDB3868, PRJEB6262, PRJEB21171, PRJNA211153, PRJDB4303, PRJEB5004, and PRJEB15503. *E. coli* ST131 sequence type is associated with multidrug resistance [12, 13]. The analysis results for *E. coli* are reported in the main manuscript.

3.1 Data acquisition

SNPs and indels were called using the NGS pipeline embedded in the *MutaNET* toolbox. We used the *S. aureus* strain NCTC 8325 as reference genome. Whole genome sequencing (WGS) paired-end reads were provided by the Helmholtz Centre for Infection Research (HZI), whereas the bacterial strains were isolated from patients that were treated at the University of Saarland Medical Center between 2010 and 2011, see [1].

Genomic information for strain NCTC 8325 was taken from the AureoWiki database [14]. This data set provided information on locus tag, gene name, strand, gene start, gene end, gene length, DNA, and protein sequences as well as operons, regulators, and accession numbers for several databases (NCBI Genbank [15], UniProtKB [16], Pfam [17]). To compare different genomic regions, we defined the promoter region as the 100 base pair sequence directly upstream of the coding region start. To investigate the effect of mutations in TFBSs, specific TF-based gene regulatory information, such as multiple sequence alignments of the TF binding sequence in different TFBSs for NCTC 8325 and similar *S. aureus* strains, was retrieved from RegPrecise [18]. Additional regulatory data was collected from the literature and the AureoWiki data set.

Information on antibiotic resistance genes was taken from the *PATRIC* database [19], which integrates the *ARDB* [20] and the *CARD* [21] databases. In addition, the literature was screened for further information on antibiotic resistance genes and regulators, such as MDR efflux pumps or MDR efflux regulators.

Table S1 lists the MDR efflux pumps and regulators in strain NCTC 8325, while Table S2 presents the antibiotic resistance genes. Table S3 is a summary of the regulators directly affecting antibiotic resistance and MDR efflux pump genes, and Table S4 lists all regulatory information taken from the literature.

Mutations in protein domains, e.g. a DNA binding domain or transmembrane helix, can affect protein function. If antibiotic resistance genes or MDR efflux pump genes are affected by mutations, this could have a large impact on antibiotic resistance. Information on protein domains and regions was obtained from the UniProtKB database [16]. The data set included the start and end positions of a domain or region within the protein, as well as an annotation on the domain function.

Table S1: MDR efflux pump genes and their direct regulators in *S. aureus* NCTC 8325. Displayed are the locus tags, gene names, the gene type, the class of antibiotics against which the gene confers resistance, and literature reference(s) for each gene.

**Locus tag**	**Gene**	**Type**	**Resistance**	**Reference(s)**
SAOUHSC_00058	*norC*	MFS pump	(fluoro–) quinolones	[22, 23]
SAOUHSC_00064	*norG*	regulator	-	[24, 25, 26]
SAOUHSC_00099	*tet38*	MFS pump	tetracyclines	[20, 22, 27, 28]
SAOUHSC_00314	*mepR*	regulator	-	[21, 29, 30, 31, 32]
SAOUHSC_00315	*mepA*	MATE pump	tigecycline	[20, 22, 28, 29, 30, 31, 32, 33]
SAOUHSC_00620	*sarA*	regulator	-	[22, 34]
SAOUHSC_00647	*abcA*	ABC pump	hydrophobic $beta$–lactams	[22, 24, 34]
SAOUHSC_00694	*mgrA*	regulator	-	[21, 22, 23, 24, 26, 27, 35, 36, 37, 38]
SAOUHSC_00703	*norA*	MFS pump	(fluoro–) quinolones	[20, 22, 23, 28, 33, 35, 36, 37, 39, 40, 41, 42]
SAOUHSC_01360	*msrA*	ABC pump	macrolides, streptogramin B, telithromycin	[43, 44, 45, 46]
SAOUHSC_01419	*arlS*	regulator	-	[21, 47]
SAOUHSC_01431	*msrB*	ABC pump	macrolides, streptogramin B, elithromycin	[43, 44]
SAOUHSC_01432	*msrA*	ABC pump	macrolides, streptogramin B, telithromycin	[43, 44, 45, 46]
SAOUHSC_01448	*norB*	MFS pump	(fluoro–) quinolones	[22, 23, 24, 27, 28, 36, 48]
SAOUHSC_01592	*fur*	regulator	-	[42]
SAOUHSC_01879	*rot*	regulator	-	[49, 50]
SAOUHSC_02418	*lmrB3*	MFS pump	-	[16, 21]
SAOUHSC_02419	*sepA*	pump	acriflavine, ehtidium bromide	[27, 51]
SAOUHSC_02669	*sarZ*	regulator	-	[22, 34, 52, 53]
SAOUHSC_02700	*lmrB2*	MFS pump	-	[16, 21]
SAOUHSC_02996	*msrA*	ABC pump	macrolides, streptogramin B, telithromycin	[43, 44, 45, 46]

Table S2: Known antibiotic resistance genes in *S. aureus* NCTC 8325. Displayed are the locus tags, gene names, the class of antibiotics against which the gene confers resistance, and literature reference(s) for each gene.

**Locus tag**	**Gene**	**Resistance**	**Reference(s)**
SAOUHSC_00005	*gyrB*	(fluoro–) quinolones	[33, 54, 55, 56, 57, 58, 59]
SAOUHSC_00006	*gyrA*	(fluoro–) quinolones, novobiocin	[33, 54, 55, 56, 57, 58, 59]
SAOUHSC_00489	*folP*	sulfonamide	[21, 60]
SAOUHSC_00524	*rpoB*	rifampicin	[21, 33]
SAOUHSC_00530	*tuf*	-	[21]
SAOUHSC_00638	*sirR*	-	[21]
SAOUHSC_00646	*pbp4*	$beta$–lactams	[22, 34, 61, 62, 63]
SAOUHSC_00691	*uppP*	bacitracin	[20]
SAOUHSC_01159	*ileS*	mupirocin	[33, 64]
SAOUHSC_01351	*parE*	novobiocin	[21, 58]
SAOUHSC_01352	*parC*	(fluoro–) quinolones	[21, 57, 58]
SAOUHSC_01359	*fmtC*	oxacillin	[21, 65]
SAOUHSC_01420	*truncated–arlR*		[21]
SAOUHSC_01434	*dfrA*	trimethoprim	[33]
SAOUHSC_01467	*pbp2*	$beta$–lactams	[34, 62, 66]
SAOUHSC_01602	*malR*	-	[21]
SAOUHSC_01800	*phoP*	-	[21]
SAOUHSC_02003	*msbA*	-	[21]
SAOUHSC_02274	*vga*	streptogramin A	[33]
SAOUHSC_02530	*marR*	-	[21]
SAOUHSC_02609	*fosB*	fosfomycin	[20, 21]
SAOUHSC_02629	-	-	[21]
SAOUHSC_02636	*tcaR*	teicoplanin	[21]
SAOUHSC_02637	*tcaR*	teicoplanin	[21]
SAOUHSC_02797	-	-	[21]
SAOUHSC_02826	-	-	[21]
SAOUHSC_02961	*slyA*	-	[21]

Table S3: Direct regulation of MDR efflux pumps in *S. aureus* NCTC 8325 according to the literature. Shown are the regulator, the genes that are regulated, the type of regulation and the literature references. The regulation is labeled as effector, if the regulator can act as an activator or repressor depending on the specific conditions in the cell, or if the precise nature of the regulation is unknown.

**Regulator**	**Regulated gene**	**Regulation**	**Reference(s)**
arlS	*norA*	effector	[47]
	norB	effector	[67]
fur	*norA*	activator	[42]
mgrA	*abcA*	activator	[22, 38]
	norA	repressor	[27, 35, 36, 37, 38]
	norB	repressor	[22, 27, 36, 38]
	norC	repressor	[23]
	norG	repressor	[24, 25, 26]
	tet38	repressor	[22, 27]
mepR	*mepA*	repressor	[29, 30, 31, 32]
norG	*abcA*	repressor	[22, 24, 25]
	norB	activator	[24, 25]
	norC	repressor	[25]
rot	*abcA*	effector	[22]
	msrA	activator	[49]
sarA	*abcA*	effector	[22, 34]
sarZ	*abcA*	effector	[34]
	norB	repressor	[22, 52, 53]
	tet38	repressor	[22, 53]

Table S4: Complete regulation in *S. aureus* NCTC 8325 according to the literature with the reference(s) for each entry. Shown are the regulator, the type of regulation, and the genes that are regulated with the literature reference(s). The regulation is labeled as effector, if the regulator can act as an activator or repressor depending on the specific conditions in the cell, or if the precise nature of the regulation is unknown.

Regulator	Regulation	Regulated gene
arlS	effector	norA [47], norB [67];
cvfA	activator	sarZ [52];
czrA	repressor	czrA [25];
	in operon	czrB [25] ;
fur	activator	norA [42];
	repressor	[68]: fhuA, fhuB, fhuD, fhuD1, fhuG, sirA, sirB, sirC, sirR; [69]: sstA, sstB, sstC, sstD;
lexA	repressor	parE [16];
mgrA	activator	[22]: abcA, agrA, agrB, agrD, sarZ; [38]: abcA, alt, bioX, cap5K, cap5M, cudT, gapB, gbsA, ggt, glmS, gltB, guaA, guaB, hlgA, htrA, lip, lrgA, lukD, lukF, lukS, lytS, mfd, mnhD, mnhE, mnhF, mnhG, mvaS, nrdE, nrdF, nrdG, nuc, pbuX, prop , purB, putP, relA, rlp, rplI, rsbW, sak, sarA, pyrR, sigB, splA, splB, splC, splD, splE, splF, srtA, ssp, tcaR, tdk, xprT, ywpF; sarZ [25], sarX [26, 70];
	repressor	[38]: arcA, arcB, capB, capB, capC, carA, carB, cidA, ddh, ebh, fmtB, ,hemD, hflX, hisF, isaB, lacA, lacB, lacC, lacD, lacE, lacF, lacG, lytN, metE, moaA, modB, moeA, mraY, mrp, mtlD, mtlF, nana, norA (see also [27, 35, 36, 37]), norB (see also [22, 27, 36]), purA, pyrB, pyrC, pyrE, pyrF, uhpT, ureA, ureB, ureC, ureD, ureE, ureF, ureG, radC, sdhA, sdhB, secY, spa; norC [23], norG [24, 25, 26], sarT [26], sarV [26, 70], tet38 [22, 27];
	effector	[38]: pyrP, pyrG, opuD, scrA;
mepA	in operon	mepB [29, 31, 32];
mepR	repressor	mepA [29, 30, 31, 32], mepR [30, 32];
norG	activator	[25]: abcA (see also [22, 24]), agrA, agrB, agrD, arlS, czrR, hrcA, lexA, lytR, lytS, mgrA, norB (see also [24]), saeR, saeS, sarR, sarX, sarZ;
	repressor	norC [25];
	effector	[25]: agrG, agrH, alt, apt, atpB, cdsA, dnaI, efb, entB hemB, hemC, hemD, hemE, hemH, hemL, hld, ilvA, ipdC, manA, mobB, modA, narG, narH, narI, narJ, nirR, oppF, pbuX, rsbV, rsbW, scdA, sdhA, sdhB, sdhC, sodA;
perR	repressor	[69]: bcp, katA, trxB;
rot	activator	[50]: capA, capB, capC, capD, capE, capF, capG, capH, capI, capJ, capL, capM, capN, capO, capP, clfB (see also [49, 71]), coa (see also [49]), kdpB, kdpD, kdpE, lrgB, manA, map, plc, rplE, rplF, rplO, rplR, rplX, rpmC, rpmD, rpsE, rpsH, sarT, spa (see also [49]), sspA, sspB2, tcaR; [49]: aldH, clpL, ctpA, dhoM, dltD, epiA, fhuA, hsdR, gltL, gtaB, lysP, lytS, msrA, nrdD, nrdI, pbuX, purM, recQ, rot (see also [70]), sdrC (see also [71]), thrB, xprT; sarA [71];
	repressor	[22]: agrA, agrB, agrD; [49]: adhE, cysK, ddh, fmhC, geh , gntK, gntP, hlb , hlgB (see also [50]), lytN, mvaK2, mvaS, narG, pmi, prsA, ptsG, splF, ureA, ureB, ureC, ureD, ureE, ureF, ureG, ribD; [50]: dut, hla, hlgA, lukD, lukE, rbsK, rpsD;
	effector	abcA [22]; [49, 50]: hlgC, kdpA, kdpC, splA, splB, splC, splD, splE, sspB, sspC;
sarA	activator	[72]: agrA, agrB, agrD, aroC, capJ, crtN, dnaG, epiF, epiP, fhuD, fnbA, fnbB, gltS, hlgB, hlgC, hutG, map, metK, opp-2B, pepF, phoP, prfA, purA, queA, rpmH, sdhB, set9 tagF, uppS; [73]: clfB, entB, hlb, lrgA; [72, 73]: femA,hla, hld, sdrC, splA, splB, splD, splF;
	repressor	[72]: arcC, arcR, atl, aur, citC, clpL, fhs, glpF, gntP, gudB, isaB, lip, mutL, ndhF, nuc, pbp3 (see also [73]), purM, rocA, rocD, spa, sspB (see also [73]), sppC, thiD; rot [22], sarA [26], sarT [26, 70], sarV [26, 70], sspA [73];
	effector	abcA [22, 34], aldA [72]; [73]: clfA, eta, femB, gyrA, hysA, lrgB (see also [72]), sak;
sarR	repressor	sarA [26, 70];
sarT	repressor	hla [74];
sarZ	activator	[52]: agrA (see also [22]), agrB (see also[22]), agrD (see also [22]), hla, hlb; [53]: efb, hisC, isdC, nuc, pbuX, purS, pyrR, sarZ, tcaA;
	repressor	[53]: argG, argH,asd, deoD, epiE, epiF, fabG, fruA, gntK, gntR, ilvD, lacD, lrgB, lysC, malA, msmX, norB (see also [22, 52]), nrgA, ohr, pckA, pflA, pflB, scrA, tet38 (see also [22]), treC, treP;
	effector	abcA [34];
sigB	activator	[75]: arlS, asp23, cap5M, clfA, clpL, crtM, crtN, csbD, epiE, epiF, epiG, fabG, fabZ, hutG ,lysP, mtlA, mtlD, murA, mvaD, mvaK1, mvaK2, opuD, rsbV, rsbW, sarA, sigB, spoVG, truB;
	repressor	[75]: aur, citM, glpQ, hla, hlgA, hlgB, hlgC, lip, lrgA, lrgB, lukF, nuc, plc, pycA, rbsD, rbsK, sak, sodM, splA, splB, splC, splD, splE, splF, sspA, sspB, sspC;

3.2 Mutation analysis

MutaNET was applied to the *E. coli* K-12 and *S. aureus* NCTC 8325 reference strains. We used mutations called with the embedded NGS pipeline from a set of 300 genomic sequences of *E. coli* taken from the NCBI (see above) and 30 *S. aureus* strains studied in [1]. Table S5 gives an overview on the analysis results.

The distribution of transitions, transversions, and indels is in accordance with the generally higher amount of transitions due to their chemical nature. The number of missense mutations was significantly lower ($p = 0.02$) in AR genes (21.3%) compared to non-AR genes (28.4%) for *S. aureus*. AR genes are important for the survival of the strain such as multidrug-resistant efflux (MDRE) pumps that can extrude a variety of substances, including signaling molecules and metabolites that are vital for cell functioning [76]. Missense mutations might be beneficial for the strain, for example by modifying the antibiotic target in such a way that renders the antibiotic ineffective. However, missense mutations in key protein domains of AR genes could disrupt the protein function and thus decrease fitness of the strain. A decreased number of missense mutations in AR genes was observed for *E. coli* as well, although this difference was statistically not significant.

Table S5: Comparison of *E. coli* and *S. aureus* by applying *MutaNET* to the datasets described in the text and using regulatory information from RegulonDB and AureoWiki, and information on resistance genes from PATRIC and the literature. AR genes include multidrug-resistant efflux (MDRE) pumps and their direct regulators. Numbers associated with AR genes are given in brackets. Density is defined as the number of mutations per kb. $*p < 0.05$ (Wilcoxon rank-sum test, AR vs. non-AR).

	E. coli	***S. aureus***
Strains in dataset	300	30
AR genes	97	47
MDRE pumps	39	13
Direct MDRE regulators	29	8
Non-AR genes	4,468	2,929
TFs	157 (35)	38 (10)
TFBS	1,794 (113)	261 (4)
Mutations	93,204 (3,035)	18,447 (372)
Transitions [%]	73.7 (74.6)	66.9 (66.7)
Transversions [%]	25.8 (24.9)	31.8 (32.2)
Indels [%]	0.4 (0.5)	1.3 (1.1)
Synonymous [%]	80.4 (82.1)	64.6 (69.4)
Missense [%]	14.9 (13.6)	28.4 (21.3)*
Mean density synonymous	16.9 (18.9)*	4.4 (5.1)*
Mean density missense	3.8 (3.7)	2.5 (1.6)

3.3 Antibiotic resistance regulatory subnetwork

To analyze the global effect of mutations, an antibiotic resistance regulatory subnetwork of *S. aureus* was constructed, see Figure S1. A truncated antibiotic resistance regulatory subnetwork of *E. coli* K-12 is shown in the main manuscript.

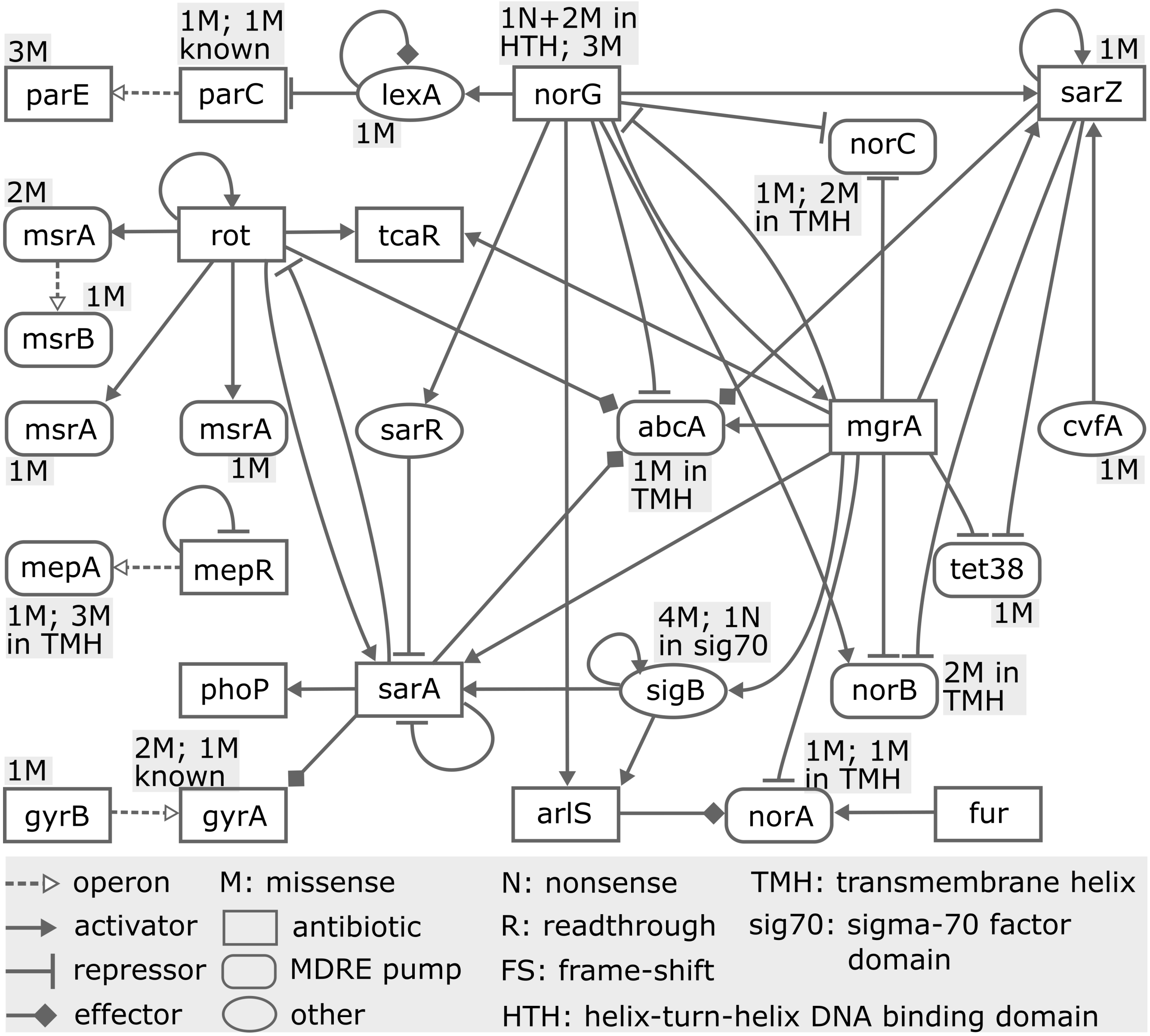

Figure S1: The antibiotic resistance regulatory subnetwork of *S. aureus* NCTC 8325 strain.

Concerning *S. aureus*, the regulator *norG* plays a key role in the regulation of the multidrug-resistant efflux (MDRE) pump. We observed a nonsense mutation K5Stop at the very beginning of the *NorG* DNA binding HTH domain that results in a non-functional protein with a length of four amino acids. In consequence, the regulators *mgrA*, *arlS*, *lexA*, *sarR*, *sarZ*, and *arlS*, as well as the MDR efflux pump *norB* would no longer be activated by *norG*. *MgrA* represses the MDR efflux pumps *norA*, *norB*, *norC* and *tet38*, while also activating the regulators *sarZ* and *sigB*, as well as the MDR efflux pump *abcA*. *SarZ* represses *norB* and *tet38*. *SarA* is indirectly regulated by *norG* via *mgrA*, *sarR* and *sigB* and in turn represses *rot* that activates the MDR efflux pumps *msrA* and *msrB*. This could result in the over-expression of the MDR efflux pumps *norA*, *norC*, *tet38*, and *abcA*, and might indirectly affect the expression of the MDR efflux pumps *norB*, *msrA*, and *msrB*, as well as the antibiotic resistance genes *parC* and *parE*. We found two additional missense mutations, namely S28P ($Score = 0.63$) and H29Q ($Score = 0.64$) in the DNA binding domain of *norG*, which could disrupt the regulatory function of *norG* sufficiently strong to cause a similar effect as described above. Note that this $Score$ was calculated as stated in the main

manuscript.

The MATE MDR efflux pump *mepA* is repressed by the regulator *mepR* and confers resistance to tigecycline, see Table S1. We found four missense mutations V167I ($Score = 0.71$), I214V ($Score = 0.69$), S332I ($Score = 0.43$), and A364T ($Score = 0.67$). The mutations I214V, S332I, and A364T are located in transmembrane helices and could thus impact the efflux function and resistance to tigecycline.

The ABC MDR efflux pump *msrA* is expressed at three different loci in the NCTC 8325 genome, one of which is part of an operon with the ABC MDR efflux pump *msrB*. *MsrA* and *msrB* confer resistance to macrolides, streptogramin B and telithromycin, see Table S1. Figure S1 shows that *MsrA* and the *msrA*–*msrB* operon are activated by the global regulator *rot*. *MsrB* may contain a L84V missense mutation with a substitution score of $Score = 0.6$ located in the methionine–R–sulfoxide reductase (MsrB) domain. The *msrA* locus *SAOUHSC_02996* may contain the missense mutations Q86E ($Score = 0.69$), Q79R ($Score = 0.59$), and A2V ($Score = 0.6$) with Q86E and Q79R mutations located in the peptide methionine sulfoxide reductase (PMSR) domain. These mutation could affect the transport function of *msrA* and *msrB* and thus impact resistance to macrolides, telithromycin, and streptogramin B.

The DNA gyrase subunit A gene, *gyrA*, confers resistance to fluoroquinolones (compare to Table S2). It is part of an operon with the DNA gyrase subunit B gene, *gyrB*, that is regulated by *sarA*. We detected a mutation in the *gyrA* promoter as well as three missense mutations S84L ($Score = 0.37$), A457T ($Score = 0.67$), and L501F ($Score = 0.6$). The S84L mutation was reported as causing resistance to fluoroquinolones in the UniProtKB data set. The UniProtKB data set also contains the fluoroquinolone resistance mutations S84A and E88K.

The RNA–polymerase sigma factor *sigB* activates the regulators *arlS* and *sarA*. A nonsense mutation Q236Stop with substitution score of $Score = 0.92$ was reported in the RNA–polymerase sigma 70 domain. This could result in loss of the domain functionality and thus decrease the ability to activate *arlS* and *sarA*. The loss of *arlS* and *sarA* could affect the expression of the MDR efflux pump *norA* and indirectly that of *msrA* and *msrB*.

In summary, mutations can have a large global effect when considering the complex GRN of an organism. The comprehensive summary of mutations visualized within the underlying GRN provided by *MutaNET* can greatly aid in the understanding and deciphering of bacterial resistance.

4 Mutation analysis across species

Applying *MutaNET* enables the analysis of mutations across various species to find similar resistance mechanisms. We found several mutations in the genes *parC* and *gyrA* for both, *E. coli* and *S. aureus*. The genes *parC* and *gyrA* were found to be associated with antibiotic resistance to (fluoro)quinolones, see Table S6.

Table S6: Mutations in *E. coli* and *S. aureus* parC and gyrA genes. ”yes” indicates that the mutation was reported, ”no” otherwise. All mutations provided by the UniProt database were associated with resistance to (fluoro)quinolones.

Gene	Protein	Organism	Mutation	*UniProt*	*MutaNET*
parC	DNA topoisomerase	*E. coli*	S80L	yes	no
			S80I	no	yes
			S80R	no	yes
			E84K	yes	no
			E84P	yes	no
			E84V	no	yes
		S. aureus	S80F	yes	yes
			S80Y	yes	no
			E84K	yes	no
			E84L	yes	no
gyrA	DNA gyrase	*E. coli*	S83A	yes	no
			S83L	yes	yes
			S83W	yes	no
			D87N	yes	yes
			D87V	yes	no
			D87G	no	yes
		S. aureus	S84L	yes	yes
			S84A	yes	no
			E88K	yes	no

This finding suggests a similar resistance mechanism on these *E. coli* and *S. aureus* strains involving *parC* and *gyrA* genes. Some of the reported mutations are known resistance mutations, whereas the other mutations reported by *MutaNET* are candidate resistance mutations. A multiple sequence alignment of *parC* and *gyrA* genes highlighting the mutated positions can be found in Figure S2.

E. coli K-12: parC (1), gyrA (3); *S. aureus* NCTC 8325: parC (2), gyrA (4)

```
       ::***: ****:*:*******: *:::* ::* :. *: *:: :** *: *. .  *
(1) 57 SAKFKKSARTVGDVLGKYHPHGDSACYEAMVLMAQPFSYRYPLVDGQGNWGAPDDPKSFA 116
(2) 57 DKNFRKSAKTVGDVIGQYHPHGDSSVYEAMVRLSQDWKLRHVLIEMHGNNGSIDN-DPPA 115
(3) 60 NKAYKKSARVVGDVIGKYHPHGDSAVYDTIVRMAQPFSLRYMLVDGQGNFGSIDG-DSAA 118
(4) 61 DKSYKKSARIVGDVMGKYHPHGDSSIYEAMVRMAQDFSYRYPLVDGQGNFGSMDG-DGAA 119
```

Figure S2: Multiple sequence alignment of *E. coli* and *S. aureus* genes *parC* and *gyrA* highlighting the positions of reported resistance and candidate resistance mutations. See also Table S6.

References

[1] M. Hamed, D. P. Nitsche-Schmitz, U. Ruffing, M. Steglich, J. Dordel, D. Nguyen, J. H. Brink, G. S. Chhatwal, M. Herrmann, U. Nubel, V. Helms, and L. von Muller. Whole genome sequence typing and microarray profiling of nasal and blood stream methicillin-resistant Staphylococcus aureus isolates: Clues to phylogeny and invasiveness. Infect. Genet. Evol., 36:475–482, Dec 2015.

[2] H. Li and R. Durbin. Fast and accurate short read alignment with Burrows-Wheeler transform. Bioinformatics, 25(14):1754–1760, Jul 2009.

[3] H. Li, B. Handsaker, A. Wysoker, T. Fennell, J. Ruan, N. Homer, G. Marth, G. Abecasis, R. Durbin, and 1000 Genome Project Data Processing Subgroup. The Sequence Alignment/Map format and SAMtools. Bioinformatics, 25(16):2078–2079, Aug 2009.

[4] H. Li. A statistical framework for SNP calling, mutation discovery, association mapping and population genetical parameter estimation from sequencing data. Bioinformatics, 27(21):2987–2993, Nov 2011.

[5] D. C. Koboldt, Q. Zhang, D. E. Larson, D. Shen, M. D. McLellan, L. Lin, C. A. Miller, E. R. Mardis, L. Ding, and R. K. Wilson. VarScan 2: somatic mutation and copy number alteration discovery in cancer by exome sequencing. Genome Res., 22(3):568–576, Mar 2012.

[6] A.R. Quinlan, I. M., and Hall. BEDTools: a flexible suite of utilities for comparing genomic features. Bioinformatics, 26(6):841–842, Mar 2010.

[7] S. Grkovic, M. H. Brown, and R. A. Skurray. Transcriptional regulation of multidrug efflux pumps in bacteria. Semin. Cell Dev. Biol., 12(3):225–237, Jun 2001.

[8] C. Melton, J. A. Reuter, D. V. Spacek, and M. Snyder. Recurrent somatic mutations in regulatory regions of human cancer genomes. Nat. Genet., 47(7):710–716, Jul 2015.

[9] K. Nishida, M. C. Frith, and K. Nakai. Pseudocounts for transcription factor binding sites. Nucleic Acids Res., 37(3):939–944, Feb 2009.

[10] A. E. Kel, E. Gossling, I. Reuter, E. Cheremushkin, O. V. Kel-Margoulis, and E. Wingender. MATCH: A tool for searching transcription factor binding sites in DNA sequences. Nucleic Acids Res., 31(13):3576–3579, Jul 2003.

[11] F. Wilcoxon. Individual Comparisons by Ranking Methods. Biometrics Bulletin, 1(6):80–83, Dec 1945.

[12] M. H. Nicolas-Chanoine, X. Bertrand, and J. Y. Madec. Escherichia coli ST131, an intriguing clonal group. Clin. Microbiol. Rev., 27(3):543–574, Jul 2014.

[13] J. R. Johnson, B. Johnston, C. Clabots, M. A. Kuskowski, and M. Castanheira. Escherichia coli sequence type ST131 as the major cause of serious multidrug-resistant E. coli infections in the United States. Clin. Infect. Dis., 51(3):286–294, Aug 2010.

[14] Aureowiki download gene specific information.

[15] D. A. Benson, K. Clark, I. Karsch-Mizrachi, D. J. Lipman, J. Ostell, and E. W. Sayers. GenBank. Nucleic Acids Res., 43(Database issue):D30–35, Jan 2015.

[16] A. Bateman, M. J. Martin, C. O'Donovan, M. Magrane, R. Apweiler, E. Alpi, R. Antunes, J. Arganiska, B. Bely, M. Bingley, C. Bonilla, R. Britto, B. Bursteinas, G. Chavali, E. Cibrian-Uhalte, A. D. Silva, M. De Giorgi, T. Dogan, F. Fazzini, P. Gane, L. G. Castro, P. Garmiri, E. Hatton-Ellis, R. Hieta, R. Huntley, D. Legge, W. Liu, J. Luo, A. MacDougall, P. Mutowo, A. Nightingale, S. Orchard, K. Pichler, D. Poggioli, S. Pundir, L. Pureza, G. Qi, S. Rosanoff, R. Saidi, T. Sawford, A. Shypitsyna, E. Turner, V. Volynkin, T. Wardell, X. Watkins, H. Zellner, A. Cowley, L. Figueira, W. Li, H. McWilliam, R. Lopez, I. Xenarios, L. Bougueleret, A. Bridge, S. Poux, N. Redaschi, L. Aimo, G. Argoud-Puy, A. Auchincloss, K. Axelsen, P. Bansal, D. Baratin, M. C. Blatter, B. Boeckmann, J. Bolleman, E. Boutet, L. Breuza, C. Casal-Casas, E. de Castro, E. Coudert, B. Cuche, M. Doche, D. Dornevil, S. Duvaud, A. Estreicher, L. Famiglietti, M. Feuermann, E. Gasteiger, S. Gehant, V. Gerritsen, A. Gos, N. Gruaz-Gumowski, U. Hinz, C. Hulo, F. Jungo, G. Keller, V. Lara, P. Lemercier, D. Lieberherr, T. Lombardot, X. Martin, P. Masson, A. Morgat, T. Neto, N. Nouspikel, S. Paesano, I. Pedruzzi, S. Pilbout, M. Pozzato, M. Pruess, C. Rivoire, B. Roechert, M. Schneider, C. Sigrist, K. Sonesson, S. Staehli, A. Stutz, S. Sundaram, M. Tognolli, L. Verbregue, A. L. Veuthey, C. H. Wu, C. N. Arighi, L. Arminski, C. Chen, Y. Chen, J. S. Garavelli, H. Huang, K. Laiho, P. McGarvey, D. A. Natale, B. E. Suzek, C. Vinayaka, Q. Wang, Y. Wang, L. S. Yeh, M. S. Yerramalla, and J. Zhang. UniProt: a hub for protein information. Nucleic Acids Res., 43(Database issue):D204–212, Jan 2015.

[17] R. D. Finn, P. Coggill, R. Y. Eberhardt, S. R. Eddy, J. Mistry, A. L. Mitchell, S. C. Potter, M. Punta, M. Qureshi, A. Sangrador-Vegas, G. A. Salazar, J. Tate, and A. Bateman. The Pfam protein families database: towards a more sustainable future. Nucleic Acids Res., 44(D1):D279–285, Jan 2016.

[18] P. S. Novichkov, A. E. Kazakov, D. A. Ravcheev, S. A. Leyn, G. Y. Kovaleva, R. A. Sutormin, M. D. Kazanov, W. Riehl, A. P. Arkin, I. Dubchak, and D. A. Rodionov. RegPrecise 3.0–a resource for genome-scale exploration of transcriptional regulation in bacteria. BMC Genomics, 14:745, 2013.

[19] A. R. Wattam, D. Abraham, O. Dalay, T. L. Disz, T. Driscoll, J. L. Gabbard, J. J. Gillespie, R. Gough, D. Hix, R. Kenyon, D. Machi, C. Mao, E. K. Nordberg, R. Olson, R. Overbeek, G. D. Pusch, M. Shukla, J. Schulman, R. L. Stevens, D. E. Sullivan, V. Vonstein, A. Warren, R. Will, M. J. Wilson, H. S. Yoo, C. Zhang, Y. Zhang, and B. W. Sobral. PATRIC, the bacterial bioinformatics database and analysis resource. Nucleic Acids Res., 42(Database issue):D581–591, Jan 2014.

[20] B. Liu and M. Pop. ARDB–Antibiotic Resistance Genes Database. Nucleic Acids Res., 37(Database issue):D443–447, Jan 2009.

[21] A. G. McArthur, N. Waglechner, F. Nizam, A. Yan, M. A. Azad, A. J. Baylay, K. Bhullar, M. J. Canova, G. De Pascale, L. Ejim, L. Kalan, A. M. King, K. Koteva, M. Morar, M. R. Mulvey, J. S. O'Brien, A. C. Pawlowski, L. J. Piddock, P. Spanogiannopoulos, A. D. Sutherland, I. Tang, P. L. Taylor, M. Thaker, W. Wang, M. Yan, T. Yu, and G. D. Wright. The comprehensive antibiotic resistance database. Antimicrob. Agents Chemother., 57(7):3348–3357, Jul 2013.

[22] R. A. Villet, Q. C. Truong-Bolduc, Y. Wang, Z. Estabrooks, H. Medeiros, and D. C. Hooper. Regulation of expression of abcA and its response to environmental conditions. J. Bacteriol., 196(8):1532–1539, Apr 2014.

[23] Q. C. Truong-Bolduc, J. Strahilevitz, and D. C. Hooper. NorC, a new efflux pump regulated by MgrA of Staphylococcus aureus. Antimicrob. Agents Chemother., 50(3):1104–1107, Mar 2006.

[24] Q. C. Truong-Bolduc and D. C. Hooper. The transcriptional regulators NorG and MgrA modulate resistance to both quinolones and beta-lactams in Staphylococcus aureus. J. Bacteriol., 189(8):2996–3005, Apr 2007.

[25] Q. C. Truong-Bolduc, P. M. Dunman, T. Eidem, and D. C. Hooper. Transcriptional profiling analysis of the global regulator NorG, a GntR-like protein of Staphylococcus aureus. J. Bacteriol., 193(22):6207–6214, Nov 2011.

[26] A. Ballal, B. Ray, and A. C. Manna. sarZ, a sarA family gene, is transcriptionally activated by MgrA and is involved in the regulation of genes encoding exoproteins in Staphylococcus aureus. J. Bacteriol., 191(5):1656–1665, Mar 2009.

[27] Q. C. Truong-Bolduc, P. M. Dunman, J. Strahilevitz, S. J. Projan, and D. C. Hooper. MgrA is a multiple regulator of two new efflux pumps in Staphylococcus aureus. J. Bacteriol., 187(7):2395–2405, Apr 2005.

[28] J. Handzlik, A. Matys, and K. Kie?-Kononowicz. Recent Advances in Multi-Drug Resistance (MDR) Efflux Pump Inhibitors of Gram-Positive Bacteria S. aureus. Antibiotics (Basel), 2(1):28–45, 2013.

[29] F. McAleese, P. Petersen, A. Ruzin, P. M. Dunman, E. Murphy, S. J. Projan, and P. A. Bradford. A novel MATE family efflux pump contributes to the reduced susceptibility of laboratory-derived Staphylococcus aureus mutants to tigecycline. Antimicrob. Agents Chemother., 49(5):1865–1871, May 2005.

[30] M. Kumaraswami, J. T. Schuman, S. M. Seo, G. W. Kaatz, and R. G. Brennan. Structural and biochemical characterization of MepR, a multidrug binding transcription regulator of the Staphylococcus aureus multidrug efflux pump MepA. Nucleic Acids Res., 37(4):1211–1224, Mar 2009.

[31] G. W. Kaatz, F. McAleese, and S. M. Seo. Multidrug resistance in Staphylococcus aureus due to overexpression of a novel multidrug and toxin extrusion (MATE) transport protein. Antimicrob. Agents Chemother., 49(5):1857–1864, May 2005.

[32] G. W. Kaatz, C. E. DeMarco, and S. M. Seo. MepR, a repressor of the Staphylococcus aureus MATE family multidrug efflux pump MepA, is a substrate-responsive regulatory protein. Antimicrob. Agents Chemother., 50(4):1276–1281, Apr 2006.

[33] S. O. Jensen and B. R. Lyon. Genetics of antimicrobial resistance in Staphylococcus aureus. Future Microbiol, 4(5):565–582, Jun 2009.

[34] G. Schrader-Fischer and B. Berger-Bachi. The AbcA transporter of Staphylococcus aureus affects cell autolysis. Antimicrob. Agents Chemother., 45(2):407–412, Feb 2001.

[35] Q. C. Truong-Bolduc, X. Zhang, and D. C. Hooper. Characterization of NorR protein, a multifunctional regulator of norA expression in Staphylococcus aureus. J. Bacteriol., 185(10):3127–3138, May 2003.

[36] Q. C. Truong-Bolduc and D. C. Hooper. Phosphorylation of MgrA and its effect on expression of the NorA and NorB efflux pumps of Staphylococcus aureus. J. Bacteriol., 192(10):2525–2534, May 2010.

[37] G. W. Kaatz, R. V. Thyagarajan, and S. M. Seo. Effect of promoter region mutations and mgrA overexpression on transcription of norA, which encodes a Staphylococcus aureus multidrug efflux transporter. Antimicrob. Agents Chemother., 49(1):161–169, Jan 2005.

[38] T. T. Luong, P. M. Dunman, E. Murphy, S. J. Projan, and C. Y. Lee. Transcription Profiling of the mgrA Regulon in Staphylococcus aureus. J. Bacteriol., 188(5):1899–1910, Mar 2006.

[39] H. Yoshida, M. Bogaki, S. Nakamura, K. Ubukata, and M. Konno. Nucleotide sequence and characterization of the Staphylococcus aureus norA gene, which confers resistance to quinolones. J. Bacteriol., 172(12):6942–6949, Dec 1990.

[40] A. A. Neyfakh, C. M. Borsch, and G. W. Kaatz. Fluoroquinolone resistance protein NorA of Staphylococcus aureus is a multidrug efflux transporter. Antimicrob. Agents Chemother., 37(1):128–129, Jan 1993.

[41] G. W. Kaatz, S. M. Seo, and C. A. Ruble. Efflux-mediated fluoroquinolone resistance in Staphylococcus aureus. Antimicrob. Agents Chemother., 37(5):1086–1094, May 1993.

[42] X. Deng, F. Sun, Q. Ji, H. Liang, D. Missiakas, L. Lan, and C. He. Expression of multidrug resistance efflux pump gene norA is iron responsive in Staphylococcus aureus. J. Bacteriol., 194(7):1753–1762, Apr 2012.

[43] F. J. Schmitz, R. Sadurski, A. Kray, M. Boos, R. Geisel, K. Kohrer, J. Verhoef, and A. C. Fluit. Prevalence of macrolide-resistance genes in Staphylococcus aureus and Enterococcus faecium isolates from 24 European university hospitals. J. Antimicrob. Chemother., 45(6):891–894, Jun 2000.

[44] J. I. Ross, E. A. Eady, J. H. Cove, W. J. Cunliffe, S. Baumberg, and J. C. Wootton. Inducible erythromycin resistance in staphylococci is encoded by a member of the ATP-binding transport super-gene family. Mol. Microbiol., 4(7):1207–1214, Jul 1990.

[45] E. Reynolds, J. I. Ross, and J. H. Cove. Msr(A) and related macrolide/streptogramin resistance determinants: incomplete transporters? Int. J. Antimicrob. Agents, 22(3):228–236, Sep 2003.

[46] E. D. Reynolds and J. H. Cove. Resistance to telithromycin is conferred by msr(A), msrC and msr(D) in Staphylococcus aureus. J. Antimicrob. Chemother., 56(6):1179–1180, Dec 2005.

[47] B. Fournier, R. Aras, and D. C. Hooper. Expression of the multidrug resistance transporter NorA from Staphylococcus aureus is modified by a two-component regulatory system. J. Bacteriol., 182(3):664–671, Feb 2000.

[48] Y. G. Kwak, Q. C. Truong-Bolduc, H. Bin Kim, K. H. Song, E. S. Kim, and D. C. Hooper. Association of norB overexpression and fluoroquinolone resistance in clinical isolates of Staphylococcus aureus from Korea. J. Antimicrob. Chemother., 68(12):2766–2772, Dec 2013.

[49] B. Said-Salim, P. M. Dunman, F. M. McAleese, D. Macapagal, E. Murphy, P. J. McNamara, S. Arvidson, T. J. Foster, S. J. Projan, and B. N. Kreiswirth. Global regulation of Staphylococcus aureus genes by Rot. J. Bacteriol., 185(2):610–619, Jan 2003.

[50] J. M. Mootz, M. A. Benson, C. E. Heim, H. A. Crosby, J. S. Kavanaugh, P. M. Dunman, T. Kielian, V. J. Torres, and A. R. Horswill. Rot is a key regulator of Staphylococcus aureus biofilm formation. Mol. Microbiol., 96(2):388–404, Apr 2015.

[51] K. Narui, N. Noguchi, K. Wakasugi, and M. Sasatsu. Cloning and characterization of a novel chromosomal drug efflux gene in Staphylococcus aureus. Biol. Pharm. Bull., 25(12):1533–1536, Dec 2002.

[52] C. Kaito, D. Morishita, Y. Matsumoto, K. Kurokawa, and K. Sekimizu. Novel DNA binding protein SarZ contributes to virulence in Staphylococcus aureus. Mol. Microbiol., 62(6):1601–1617, Dec 2006.

[53] P. R. Chen, S. Nishida, C. B. Poor, A. Cheng, T. Bae, L. Kuechenmeister, P. M. Dunman, D. Missiakas, and C. He. A new oxidative sensing and regulation pathway mediated by the MgrA homologue SarZ in Staphylococcus aureus. Mol. Microbiol., 71(1):198–211, Jan 2009.

[54] J. Yamagishi, T. Kojima, Y. Oyamada, K. Fujimoto, H. Hattori, S. Nakamura, and M. Inoue. Alterations in the DNA topoisomerase IV grlA gene responsible for quinolone resistance in Staphylococcus aureus. Antimicrob. Agents Chemother., 40(5):1157–1163, May 1996.

[55] E. Y. Ng, M. Trucksis, and D. C. Hooper. Quinolone resistance mutations in topoisomerase IV: relationship to the flqA locus and genetic evidence that topoisomerase IV is the primary target and DNA gyrase is the secondary target of fluoroquinolones in Staphylococcus aureus. Antimicrob. Agents Chemother., 40(8):1881–1888, Aug 1996.

[56] H. Ito, H. Yoshida, M. Bogaki-Shonai, T. Niga, H. Hattori, and S. Nakamura. Quinolone resistance mutations in the DNA gyrase gyrA and gyrB genes of Staphylococcus aureus. Antimicrob. Agents Chemother., 38(9):2014–2023, Sep 1994.

[57] F. D. Lowy. Antimicrobial resistance: the example of Staphylococcus aureus. J. Clin. Invest., 111(9):1265–1273, May 2003.

[58] M. Fujimoto-Nakamura, H. Ito, Y. Oyamada, T. Nishino, and J. Yamagishi. Accumulation of mutations in both gyrB and parE genes is associated with high-level resistance to novobiocin in Staphylococcus aureus. Antimicrob. Agents Chemother., 49(9):3810–3815, Sep 2005.

[59] L. Ferrero, B. Cameron, and J. Crouzet. Analysis of gyrA and grlA mutations in stepwise-selected ciprofloxacin-resistant mutants of Staphylococcus aureus. Antimicrob. Agents Chemother., 39(7):1554–1558, Jul 1995.

[60] P. Huovinen. Resistance to trimethoprim-sulfamethoxazole. Clin. Infect. Dis., 32(11):1608–1614, Jun 2001.

[61] G. Memmi, S. R. Filipe, M. G. Pinho, Z. Fu, and A. Cheung. Staphylococcus aureus PBP4 is essential for beta-lactam resistance in community-acquired methicillin-resistant strains. Antimicrob. Agents Chemother., 52(11):3955–3966, Nov 2008.

[62] T. A. Leski and A. Tomasz. Role of penicillin-binding protein 2 (PBP2) in the antibiotic susceptibility and cell wall cross-linking of Staphylococcus aureus: evidence for the cooperative functioning of PBP2, PBP4, and PBP2A. J. Bacteriol., 187(5):1815–1824, Mar 2005.

[63] U. U. Henze and B. Berger-Bachi. Staphylococcus aureus penicillin-binding protein 4 and intrinsic beta-lactam resistance. Antimicrob. Agents Chemother., 39(11):2415–2422, Nov 1995.

[64] A. S. Lee, Y. Gizard, J. Empel, E. J. Bonetti, S. Harbarth, and P. Francois. Mupirocin-induced mutations in ileS in various genetic backgrounds of methicillin-resistant Staphylococcus aureus. J. Clin. Microbiol., 52(10):3749–3754, Oct 2014.

[65] H. Komatsuzawa, K. Ohta, T. Fujiwara, G. H. Choi, H. Labischinski, and M. Sugai. Cloning and sequencing of the gene, fmtC, which affects oxacillin resistance in methicillin-resistant Staphylococcus aureus. FEMS Microbiol. Lett., 203(1):49–54, Sep 2001.

[66] C. J. Hackbarth, T. Kocagoz, S. Kocagoz, and H. F. Chambers. Point mutations in Staphylococcus aureus PBP 2 gene affect penicillin-binding kinetics and are associated with resistance. Antimicrob. Agents Chemother., 39(1):103–106, Jan 1995.

[67] S. S. Costa, M. Viveiros, L. Amaral, and I. Couto. Multidrug Efflux Pumps in Staphylococcus aureus: an Update. Open Microbiol J, 7:59–71, 2013.

[68] A. Xiong, V. K. Singh, G. Cabrera, and R. K. Jayaswal. Molecular characterization of the ferric-uptake regulator, fur, from Staphylococcus aureus. Microbiology (Reading, Engl.), 146 (Pt 3):659–668, Mar 2000.

[69] M. J. Horsburgh, E. Ingham, and S. J. Foster. In Staphylococcus aureus, fur is an interactive regulator with PerR, contributes to virulence, and Is necessary for oxidative stress resistance through positive regulation of catalase and iron homeostasis. J. Bacteriol., 183(2):468–475, Jan 2001.

[70] A. L. Cheung, K. A. Nishina, M. P. Trotonda, and S. Tamber. The SarA protein family of Staphylococcus aureus. Int. J. Biochem. Cell Biol., 40(3):355–361, 2008.

[71] H. Y. Hsieh, C. W. Tseng, and G. C. Stewart. Regulation of Rot expression in Staphylococcus aureus. J. Bacteriol., 190(2):546–554, Jan 2008.

[72] P. M. Dunman, E. Murphy, S. Haney, D. Palacios, G. Tucker-Kellogg, S. Wu, E. L. Brown, R. J. Zagursky, D. Shlaes, and S. J. Projan. Transcription profiling-based identification of Staphylococcus aureus genes regulated by the agr and/or sarA loci. J. Bacteriol., 183(24):7341–7353, Dec 2001.

[73] A. L. Cheung, A. S. Bayer, G. Zhang, H. Gresham, and Y. Q. Xiong. Regulation of virulence determinants in vitro and in vivo in Staphylococcus aureus. FEMS Immunol. Med. Microbiol., 40(1):1–9, Jan 2004.

[74] K. A. Schmidt, A. C. Manna, S. Gill, and A. L. Cheung. SarT, a repressor of alpha-hemolysin in Staphylococcus aureus. Infect. Immun., 69(8):4749–4758, Aug 2001.

[75] M. Bischoff, P. Dunman, J. Kormanec, D. Macapagal, E. Murphy, W. Mounts, B. Berger-Bachi, and S. Projan. Microarray-based analysis of the Staphylococcus aureus sigmaB regulon. J. Bacteriol., 186(13):4085–4099, Jul 2004.

[76] J. Sun, Z. Deng, and A. Yan. Bacterial multidrug efflux pumps: mechanisms, physiology and pharmacological exploitations. Biochem. Biophys. Res. Commun., 453(2):254–267, Oct 2014.
